# Systemic contact dermatitis following spinal cord stimulation in a patient with complex regional pain syndrome: A case report

**DOI:** 10.1097/MD.0000000000042460

**Published:** 2025-05-16

**Authors:** Jimyeong Jeong, Yeong Seung Ko, Hye Rim Kwon, Hyun Kyoung Lim, Na Eun Kim

**Affiliations:** a Department of Anesthesiology and Pain Medicine, Inha University Hospital, Inha University School of Medicine, Incheon, South Korea.

**Keywords:** contact dermatitis, hypersensitivity reaction, spinal cord stimulation

## Abstract

**Rationale:**

Spinal cord stimulation (SCS) has been widely used since the 1960s to treat intractable pain. However, hypersensitivity related to inserted devices such as implantable pulse generators, leads, and electrode contacts has rarely been reported as a side effect. We report a case of systemic contact dermatitis following SCS insertion in a patient with complex regional pain syndrome and suggest preventive and therapeutic strategies.

**Patient’s concerns:**

A 50-year-old man was diagnosed with complex regional pain syndrome due to persistent severe pain following surgery for a left ankle fracture and sprain. The patient had an SCS inserted because his leg pain was not controlled despite medication and nerve block for several years, and he was discharged without any side effects.

**Diagnoses:**

A dermatologist diagnosed the patient with contact dermatitis and prescribed medications.

**Outcomes:**

Itching worsened near the arms, neck, and scrotum; hence, 4 mg methylon and 0.3% difuco ointment (difucortolone valerate) were additionally prescribed. The patient was undergoing continuous treatment in the pain and dermatology department for these symptoms.

**Lessons:**

Hypersensitivity after SCS insertion is difficult to treat and may require the removal of the insertion device. Therefore, the patient’s medical history and screening tests, such as patch tests, are important before the procedure.

## 1. Introduction

Since the 1960s, neuromodulation devices have been widely used to treat patients with intractable chronic neuropathic pain. Side effects after the procedure include infection, bleeding, lead migration, malfunction of the generator, and rare side effects such as hypersensitivity and allergic reactions related to the insertion devices.^[1]^

The cause of contact dermatitis after the procedure was associated with the lead and 99.32% titanium used, with traces of Fe, Ni, Sn, Sb, Mo, Mn, and Ni acting as potential antigens. However, no clear cause was identified. Treatment with topical corticosteroids or device removal has also been reported.^[2]^

The patient’s skin symptoms are systemic, and the change in itching according to the intensity of the spinal cord stimulator cannot be explained by simple contact hypersensitivity alone.

According to Davidson et al, the side effects of contact dermatitis after the procedure are the responses of 100 neurons in the spinothalamic tract (STT). This is because of a common neural pathway between histamine and evoked pruritus. However, there is a lack of research or data that proves that spinal cord stimulators are the cause of neural itching.

Systemic contact dermatitis that occurred after spinal cord stimulation (SCS) insertion was discussed from the above 2 perspectives.

## 2. Case presentation

A 50-year-old man was diagnosed with an ankle sprain after a left ankle injury in April 2021. The patient was treated conservatively at another hospital before visiting our pain clinic because of persistent and aggravated pain and swelling. He had no significant medical history, was 167 cm tall, and weighed 63.1 kg. The patient was diagnosed with complex regional pain syndrome (CRPS) type 1 based on symptomatic and objective examinations.

Treatment using 3 lumbar neurolysis procedures with alcohol and nerve blocks was insufficient for pain control. Hence, after trial implantation on October 27, 2023, permanent SCS was performed on October 31, 2023. After the patient entered the operating room, blood pressure, oxygen saturation, and electrocardiography were monitored, and diagnostic implantation was performed in the prone position under local anesthesia. The needle was advanced into the L1–L2 interlaminar space, The 8-lead catheter tip level was placed on the T11 vertebral upper endplate. Appropriate signal transmission was confirmed after connecting with an external trial stimulator. Five days after the procedure, no side effects were observed and a pulse generator was inserted into the lower right quadrant of the subcutaneous abdomen under general anesthesia. No adverse effects were observed after the procedure, and the patient was discharged from the hospital 10 days later after suture removal.

**Figure 1.**
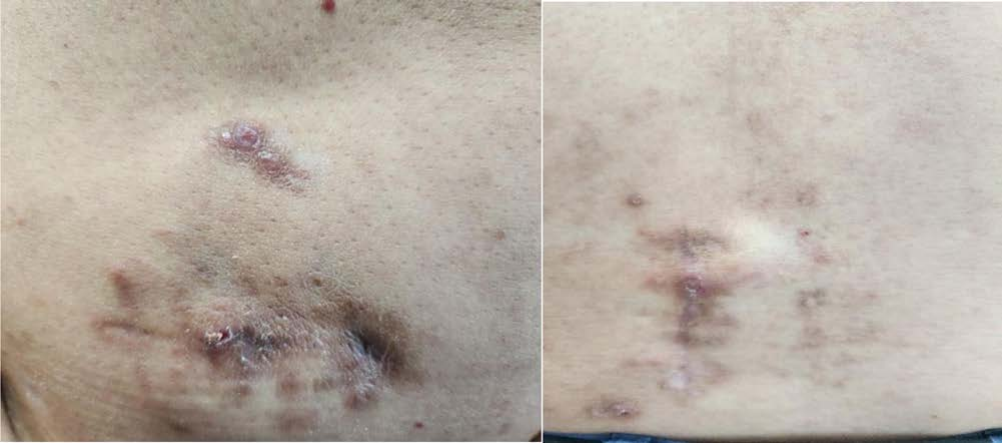
Insertion sites for spinal cord stimulation in the abdomen and back.

**Figure 2.**
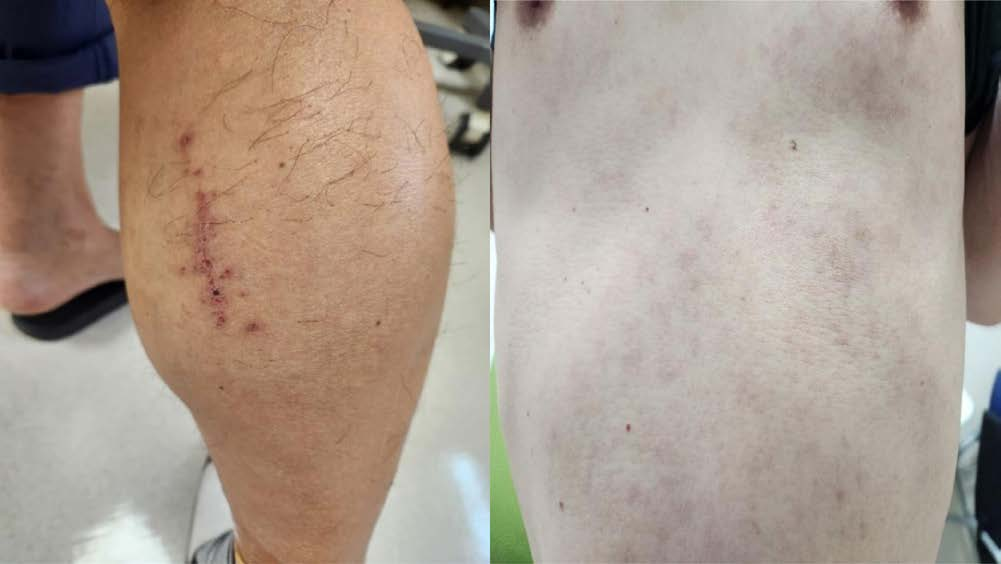
Skin irritation on the contralateral limb and abdomen.

One month after the procedure, the patient experienced itching and erythema around the vesicle because of the taping; therefore, he consulted a dermatologist (Figs. 1 and 2). Irritant contact dermatitis was diagnosed, and treatment was initiated with an antihistamine (Allegra 180 mg) and a barrier dermal lotion (moisturizer). Photo-chemotherapy was administered. This was followed by itching of the scalp, rash, and wheel at the sites of intravenous injection and nerve block. The itching worsened near the arms, neck, and scrotum; hence, 4 mg of methylon and 0.3% difuco ointment (difluorochlorovalerate) were additionally prescribed. The patient was undergoing continuous treatment in the pain and dermatology department for these symptoms. After administering Janus kinase 1 (JAK1) inhibitors (upadacitinib) 15 mg. It was first approved in Korea in 2020 as a treatment for rheumatoid arthritis, and its range of adaptations is expanding to atopic dermatitis and inflammatory bowel disease. Side effects include upper respiratory tract infection, neutropenia, anemia, and increased liver enzyme levels, so upadacitinib 15 mg is prescribed once a day for these patients to closely monitor.

## 3. Discussion and conclusion

This case is a report on contact dermatitis that occurred after SCS insertion in patients with CRPS and was examined from 2 perspectives: direct contact hypersensitivity due to the spinal cord stimulator device and neural mechanism of itching.

Allergic contact dermatitis associated with metal ions is known to occur by forming protein complexes called T-cell epitopes. Natural killer T cells and dendritic epidermal T cells are known to be important effectors in contact dermatitis and are known to induce various interleukin activities. In addition, nickel directly induces TLR4 dimerization and nuclear factor kappa B activation, and activates inflammasomes to induce interleukin (IL)-1β production, causing contact hypersensitivity.^[3]^ The insertion material of SCS was used as a contact antigen, and the itching was relieved after the use of antiallergic drugs, or depending on the severity, the removal of the SCS devices.

Itching is a process in which various stimuli are transmitted to the cerebral cortex and hypothalamus through the STT via peripheral sensory nerve A δ and C unmyelinated nerve fibers. STT neurons, located in the deep dorsal horn, are sensitive to mechanical stimulation, intradermal capsaicin, and heat.

According to Mochizuki et al,^[4]^ several studies have indicated that the relationship between itching and pain is minimal, as they share the same neurotransmission pathway but exhibit different magnitudes of activity in the thalamus.^[5]^

Previous cases have been skin lesions at the SCS insertion site, but in this case, nerve block sites for pain control and operation sites after the procedure were also accompanied by skin lesions, which were described as systemic contact dermatitis.

Itching interacts with sensory nerves in the spinal cord and transmits a response. Neurons in the spinal cord (gastrin-releasing peptide) simultaneously accept pain and itching stimuli but respond selectively through enkephalin signaling or inhibitory interneurons.^[6]^

The occurrence of dermatitis after spinal cord stimulator insertion is rare, and most of them are described as allergic association with spinal cord stimulator insertion sites.

Further research is needed to find out the association of basic helix-loop-helix (BHLHB5+) inhibitory interneurons by spinal cord stimulator, excessive itching because of pain suppression, and excessive scratching of primate STT neurons.^[7]^ If the spinal cord stimulator actually causes nerve itching, then only removal of the stimulator may be the cure, so further neurological and immunological studies are needed.

JAK1 inhibitors (upadacitinib) have been used for several conditions that cause refractory itching since Food and Drug Administration approval in 2022.

Upadacitinib is a selective JAK inhibitor known to inhibit IL-6 and IL-7 cytokine signaling through phosphorylation. Therefore, it has recently been used as an important cytokine modulator in many inflammatory diseases. Upadacitinib is a competitive inhibitor of adenosine triphosphate and can inhibit the JAK pathway. JAK pathway has been shown to relieve itching after drug use, so studies are needed on potentiating neuronal responses associated with other itch mediators. JAK pathway is a mechanism that inhibits scratching behavior by increasing intracellular calcium concentration by acting on TRPV1 and has recently been used as a treatment for refractory atopic dermatitis.^[8]^

Upadacitinib is a drug that needs to be controlled by closely monitoring liver dysfunction and changes in blood levels, as cases of a rapid increase in white blood cells and increased infections have been reported because of the influence of the immune system.^[9]^

In addition, in Australia, drugs carry a black box warning and are provided to patients after informed consent. Therefore, in Korea, it has been used for various intractable diseases since Food and Drug Administration approval in 2022, but it is thought that sufficient caution and prior consent are required according to the level of risk.

Since the 1990s, SCS, dorsal root ganglion, and peripheral nerve stimulation have been used to treat patients with CRPS and intractable pain despite pharmacotherapy, nerve blocks, or physical therapy.^[10]^

SCS is a method of activating the pain inhibition pathway through stimulation of the spinal dorsal horn by inserting an electrode and a pulse generator that provides electrical stimulation signals through the epidural space.^[11]^ The SCS mechanism is explained by gate control theory, spinal cord thalamus conduction block, supraspinal inhibition, and inhibitory neurotransmitter activation.^[12]^

The relationship between itching and pain after SCS insertion has been explained through a common pathway to STT but with a different mechanism of action.^[5]^ It is described as an allergic reaction caused by titanium or nickel, the materials used in SCS electrodes, or pulse generators.^[13]^

The SCS consists of an implantable pulse generator (IPG), a lead, and an electrode that is in contact with the ends of the lead. The case surrounding the IPG was composed of more than 90% titanium mixed with small amounts of iron, nickel, tin, antimony, and molybdenum.^[2]^ The lead is a cable insulated with silicone or polyurethane.

The components related to the insertion device are as follows (Table 1).

**Table 1:** Spinal cord stimulation components.

Material	Abbott (proclaim)	Boston Scientific (precision)	Nerro (senza)	Medtronics
Metals				
Titanium	Yes	IPG case	Yes	Case, connector block
Platinum	Electrode	Distal contacts on the percutaneous lead	Electrode	
Stainless steel	Yes	IPG connector block	Yes	
Organics				
Polyurethane	IPG, lead insulation	IPG port plug		Neurostimulator plug
Silicone	IPG, paddle	Suture sleeves		Connector block, Grommets, seals
Polysulfone	Port plug			Connector block
Fluoropolymer				Insulation

IPG = implantable pulse generator.

Manoušek et al^[14]^ reported the use of aluminum, nickel, and platinum as materials for SCS devices, which later caused hypersensitivity to silicone, polyurethane, and titanium. Hypersensitivity associated with neuromodulation devices has been reported in approximately 13 patients. Reported allergens include polyurethane, nickel, platinum, and silicone.^[15]^

Symptoms were reported to occur between 8 days and 3 years after the procedure. Hypersensitivity was defined as skin symptoms, pain, and swelling at the lead insertion site.^[16]^

Skin symptoms such as eczema, erythema, and pruritus were present. Two of the patients developed systemic symptoms.^[17]^ Hypersensitivity reactions have been reported following the removal of the neuromodulation device or the use of topical steroids.^[18]^

Since no clear and effective treatment exists, taking a history of metal allergy and performing an appropriate patch test before the procedure was considered safe.

If a titanium allergy was detected through prior testing, a gold- or silicone-coated IPG was used. If an allergy to polyurethane or parylene was detected, silicone-coated components were used. Silicone-free components were used if silicone allergy was detected.^[19,20]^

The skin lesions showed symptoms of contact dermatitis not only around the area of device insertion but also at the nerve block site after the procedure. In addition to contact dermatitis caused by the insertion devices, pruritus evoked by a common spinothalamic pathway should be considered and studied in the future.

Although reports of hypersensitivity related to SCS insertion are rare, taking preventive measures based on accurate medical history and patch testing before the procedure is important. SCS removal should be considered in cases in which treatment is difficult and hypersensitivity is uncontrolled. If an allergic reaction is expected after surgery, a multidisciplinary approach between dermatologists, allergists, and medical device manufacturers is recommended.

## Author contributions

**Writing – original draft:** Jimyeong Jeong, Na Eun Kim.

**Writing – review & editing:** Jimyeong Jeong, Na Eun Kim.

**Conceptualization:** Na Eun Kim.

**Funding acquisition:** Na Eun Kim.

**Supervision:** Na Eun Kim, Hyun Kyoung Lim.

**Data curation:** Yeong Seung Ko, Hye Rim Kwon.

**Methodology:** Yeong Seung Ko.

**Project administration:** Yeong Seung Ko, Hye Rim Kwon.

**Software:** Yeong Seung Ko, Hye Rim Kwon.

**Validation:** Hye Rim Kwon.
